# Recent Advances in Strategies for Addressing Hypoxia in Tumor Photodynamic Therapy

**DOI:** 10.3390/biom12010081

**Published:** 2022-01-05

**Authors:** Liang Hong, Jiangmin Li, Yali Luo, Tao Guo, Chenshuang Zhang, Sha Ou, Yaohang Long, Zuquan Hu

**Affiliations:** 1 Key Laboratory of Environmental Pollution Monitoring and Disease Control of Ministry of Education, Immune Cells and Antibody Engineering Research Center of Guizhou Province, School of Biology and Engineering, Guizhou Medical University, Guiyang 550025, China; hongliang@gmc.edu.cn (L.H.); lijiangmin@stu.gmc.edu.cn (J.L.); luoyali@stu.gmc.edu.cn (Y.L.); guotao@stu.gmc.edu.cn (T.G.); ousha@gmc.edu.cn (S.O.); longyaohang@gmc.edu.cn (Y.L.); 2 Key Laboratory of Optoelectronic Devices and Systems of Ministry of Education and Guangdong Province, College of Optoelectronic Engineering, Shenzhen University, Shenzhen 518060, China; 1950453008@email.szu.edu.cn

**Keywords:** nanomaterials, photodynamic therapy, hypoxia, oxygen, tumor

## Abstract

Photodynamic therapy (PDT) is a treatment modality that uses light to target tumors and minimize damage to normal tissues. It offers advantages including high spatiotemporal selectivity, low side effects, and maximal preservation of tissue functions. However, the PDT efficiency is severely impeded by the hypoxic feature of tumors. Moreover, hypoxia may promote tumor metastasis and tumor resistance to multiple therapies. Therefore, addressing tumor hypoxia to improve PDT efficacy has been the focus of antitumor treatment, and research on this theme is continuously emerging. In this review, we summarize state-of-the-art advances in strategies for overcoming hypoxia in tumor PDTs, categorizing them into oxygen-independent phototherapy, oxygen-economizing PDT, and oxygen-supplementing PDT. Moreover, we highlight strategies possessing intriguing advantages such as exceedingly high PDT efficiency and high novelty, analyze the strengths and shortcomings of different methods, and envision the opportunities and challenges for future research.

## 1. Introduction

Cancer is one of the leading causes of disease-associated death in the world. Currently, traditional cancer treatment approaches face bottlenecks such as inadequate efficiency for destroying tumor and severe damage to normal tissues due to low selectivity. For example, surgery may cause severe influence on organ functions. Chemotherapy and radiotherapy are always accompanied by serious drug resistance and severe side effects, which lead to a serious influence on the life quality of patients [1]. Compared with traditional treatments, photodynamic therapy (PDT) provides advantages including high targeting ability, noninvasiveness, negligible side effects, maximal preservation of tissue functions, desired convenience, and high patient compliance [2]. Tumor PDT is a treatment modality that relies on cytotoxic reactive oxygen species (ROS) generated with a photosensitizer under light irradiation to kill cancer cells. It can be categorized into type I and type II PDT. The common PDT is type II PDT, as most photosensitizers are type II photosensitizers. Type II PDT kills tumor cells through the conversion of molecular oxygen to cytotoxic singlet oxygen (^1^O_2_, a type of ROS) with a photosensitizer, when it is delivered to the tumor and irradiated by light. In spite of enormous advantages, the application of PDT still faces great challenges, including the lack of effective photosensitizers, limited biological tissue penetration depth of light, and a harsh tumor microenvironment (TME) featured by severe hypoxia and other related characteristics [3].

Hypoxia is a salient and important feature of solid tumors. The typical oxygen pressure level in the tumor hypoxic area is below 2.5 mm Hg, whereas in normal tissues, the oxygen pressure suits metabolic requirements, and is usually above 40 mm Hg [4,5]. The hypoxia is derived from the rapid tumor cell proliferation and abnormal tumor vascular system. It is widely recognized that hypoxia promotes tumor angiogenesis, metastasis, and resistance to many therapies including chemotherapy, radiotherapy, and PDT [6]. In particular, hypoxia impedes type II PDT efficiency severely, since oxygen is a critical component for type II PDT. What is worse, hypoxia is aggravated during the type II PDT process due to the oxygen consumption and vascular closure. In addition, hypoxia inhibits the immune response triggered by type II PDT [7]. In a word, hypoxia is a major stumbling block of type II PDT.

Currently, the development of strategies for addressing tumor hypoxia in type II PDT is a focus for cancer research, and novel approaches and attractive nanomaterials are continuously emerging. This review summarizes the cutting-edge progress on approaches to address tumor hypoxia and improve PDT, analyzes the strengths and shortcomings of these approaches, and puts forward potential challenges and future opportunities for further research. To define clearly the scope of this article, this review focuses on the methods that directly address tumor hypoxia and advance light-activated PDT. Therefore, some excellent methods that affect hypoxia indirectly, such as suppression of hypoxia-related proteins including hypoxia inducible factor-1α (HIF-1α), or approaches that do not involve light application, such as the employment of hypoxia-activable drugs (e.g., tirapazamine [8] and banoxantrone (AQ4N)) [9], are not included in this article. The discussion on these outstanding tactics can be found in other review articles [5,10,11].

## 2. Strategies for Modulating Tumor Hypoxia in PDT

Early strategies for overcoming tumor hypoxia in type II PDT include hyperbaric oxygen breathing and inhaling pure oxygen or carbogen (95% oxygen + 5% carbon dioxide) at atmospheric pressure [12]. These methods, nontheless, have gained limited achievements due to their insufficient therapeutic efficacy and side effects such as hyperoxic seizures and barotrauma [13].

Recent strategies focus on the development of advanced nanomedicines that could address hypoxia and simultaneously improve PDT efficacy. The innovation of nanomaterials has become the core impetus for the development of PDT for hypoxic tumors. These nanomaterial-based strategies can be categorized into three domains, including oxygen-independent phototherapy, oxygen-economizing PDT, and oxygen-supplementing PDT.

### 2.1. Oxygen-Independent Phototherapy

The major merit of PDT, i.e., high tumor-targeting ability which facilitates prevention of damage to normal tissues, originates from the controllable and tunable nature of light and the cytotoxic effect of ROS. However, typical oxygen-dependent ROS production severely limits type II PDT efficiency. Therefore, therapeutic modalities which can generate cytotoxic substance other than ROS, or cytotoxic physical effect under light irradiation, might overcome oxygen dependence while keeping the high tumor specificity. Meanwhile, light-enhanced Fenton/Fenton-like reaction and type I PDT could circumvent the tumor hypoxia issue.

#### 2.1.1. Photogenerated Hole Therapy

**Figure 1:**
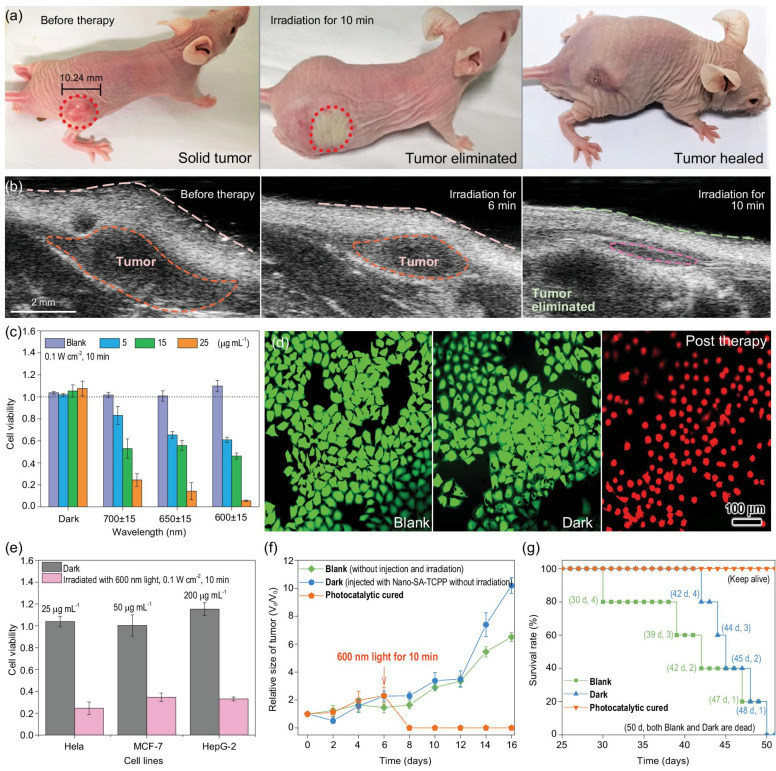
The photogenerated hole therapy with Nano-SA-TCPP. (**a**) The images of photogenerated hole therapy for tumor-bearing mice (irradiation condition: 600 nm, 0.1 W cm^−2^). (**b**) The ultrasound pictures of tumors before and after photogenerated hole therapy (irradiation condition: 600 nm, 0.1 W cm^−2^). (**c**) The viability of Hela cells with various concentrations of Nano-SA-TCPP under light irradiation. (**d**) Fluorescence images of living (green) and dead (red) Hela cells (irradiation condition: 600 nm, 0.1 W cm^−2^). (**e**) Photogenerated hole therapy on diverse cancer cell lines (irradiation condition: 600 nm, 0.1 W cm^−2^). (**f**) Relative tumor volumes of mice. (**g**) Survival rates of mice. Nano-SA-TCPP, nano-self-assembled tetra-carboxyphenyl porphyrin. Reprinted with permission from Ref. [14]. Copyright 2020 Oxford University Press.

Different from the traditional ^1^O_2_-producing PDT, a novel highly efficient phototherapy was developed by Zhang et al. based on the generation of photogenerated holes (Figure 1) [14]. Photogenerated holes are high-energy oxidation states formed on the semiconductor photocatalyst surface after excitation by light [15]. The photogenerated holes could exert a strong oxidation effect on cancer cells, causing cancer cell death. As the formation of photogenerated holes does not rely on oxygen, the treatment efficiency could be improved. Of note, an unprecedentedly high therapeutic efficiency was achieved by this treatment modality. Supramolecular photocatalysts of self-assembled tetra-carboxyphenyl porphyrin (SA-TCPP) were prepared and administrated into subcutaneous tumor-bearing mice. After irradiation with a 600 nm light of 0.1 W cm^−2^ for 10 min, the tumors (100 mm^3^) were completely eliminated. Within 10 minutes, the solid tumor site was atrophied and flattened. On the second day, the irradiated part was scarred. One week later, the scars fell off, and newly reconvened tissues were revealed. After 50 days, all the treated mice were healthy, whereas none of the untreated control group mice survived. In addition to the extraordinary therapeutic efficiency, this treatment modality showed satisfactory biosafety. This therapy opened up new possibilities for hypoxic tumor phototherapy. With further development, the photogenerated hole therapy may play a significant role in tumor treatment.

#### 2.1.2. Photoacoustic Therapy

The photoacoustic effect describes the formation of acoustic waves following absorption of light pulses. It has been utilized for causing mechanical destruction of cancer cells [16]. Because of its oxygen-independent nature, photoacoustic effect provides an effective approach for addressing hypoxia in PDT. A recent research demonstrated that under pulse laser irradiation, gadolinium(III)-phthalocyanine (GdPc) could simultaneously produce an intense acoustic effect and a large amount of ^1^O_2_. Combinatorial inhibition of tumors was detected under either normal or hypoxic conditions [17].

#### 2.1.3. Photo-Acid Therapy

**Figure 2:**
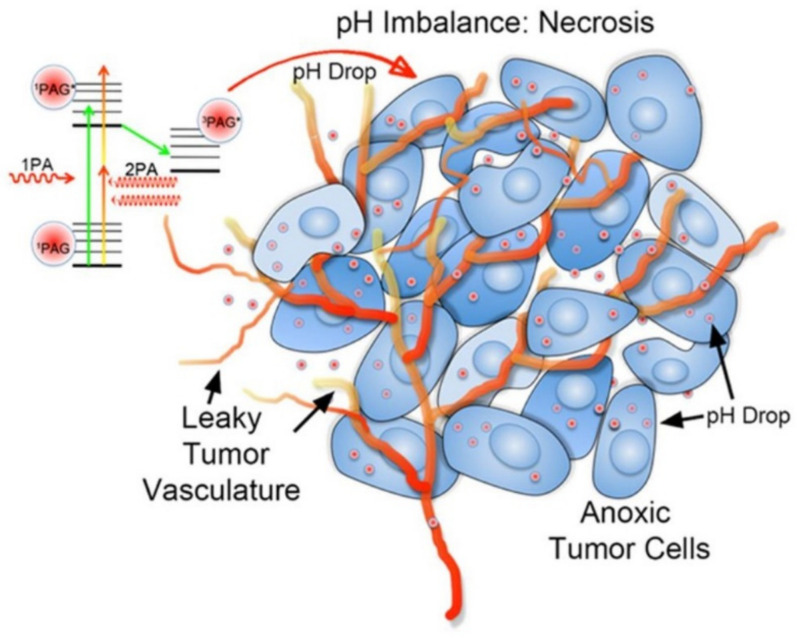
Schematic illustration of photo-acid therapy with photoacid generators (PAGs) under two-photon excitation. Adapted with permission from Ref. [18]. Copyright 2013 American Chemical Society.

In 2013, Yue et al. developed a novel photo-acid therapy to overcome hypoxia in tumor PDT treatment (Figure 2) [18]. Photoacid generators (PAGs) are molecules which could generate a pH drop when irradiated by light. Sulfonium salts were traditionally used as PAGs in lithography and cationic polymerization, and they could produce photoacid under excitation by ultraviolet or deep-ultraviolet light. However, the short wavelength limited the biological tissue penetration depth, impeding deep-seated tumor treatment. To shift the light wavelength to the visible and near-infrared (NIR) light range, Yue et al. designed and synthesized more conjugated and two-photon absorbing sulfonium salts. These sulfonium salts induced considerable cancer cell death through generating a pH drop in the cytosol under light irradiation, and showed low cytotoxicity in the dark.

Later in 2016, polyethylene glycol (PEG)-functionalized and hydrophilic silica nanoparticle-enriched PAGs were developed for increasing aqueous solubility and effectively inducing cancer cell death [19]. In 2019, a novel iridium(III)-based PAG was synthesized for mitochondria-targeted dual-mode (oxygen-independent and oxygen dependent) tumor phototherapy [20]. This iridium(III)-based PAG was able to produce photoacid under light irradiation in hypoxic environment, and its photolysis products could further sensitize ^1^O_2_ generation. Moreover, it is noteworthy that in 2019, Tian et al. constructed a new PAG which could generate large pH jumps at low concentrations [21]. This feature is important for tumor photo-acid therapy, and is worthy of further exploration. With further development, photo-acid therapy may play an important role in hypoxic tumor treatment.

#### 2.1.4. Photo-Induced Alkyl Radical Generation Therapy

**Figure 3:**
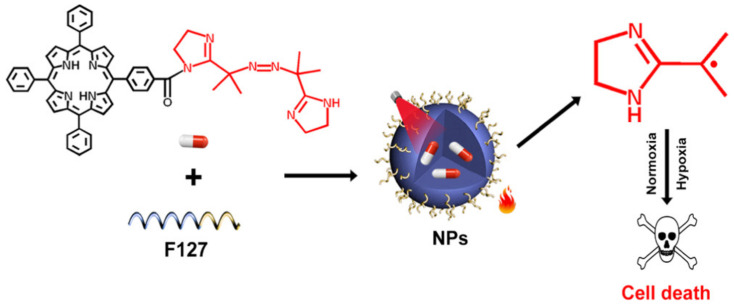
Schematic illustration of the structure of TPP-NN NPs and the mechanism of photo-induced alkyl radical generation therapy. Reprinted with permission from Ref. [23]. Copyright 2019 American Chemical Society.

Recently, a growing number of studies have focused on the oxygen-independent photo-induced alkyl radical generation for tumor treatment [22]. Alkyl radicals and ROS are both free radicals that could damage cancer cells. However, the production of alkyl radicals from azo compounds does not rely on oxygen, and could be induced by heat stimulation. In this context, in 2019, Xia et al. conjugated a photothermal porphyrin with an alkyl radical initiator to obtain a new kind of nanoparticles denoted as TPP-NN NPs (Figure 3) [23]. Under NIR light irradiation, TPP-NN NPs could split and release alkyl radicals, causing cancer cell death in both normoxic and hypoxic conditions. In vitro and in vivo studies showed that the photothermal-controlled production of alkyl radicals could exert excellent anticancer effects with negligible systemic toxicity. Similarly, another photothermal agent, semiconducting polymer nanoparticles, were combined with an alkyl radical initiator and exhibited satisfactory antitumor effects under NIR irradiation [24].

The overproduced glutathione in TME could scavenge free radicals; therefore, it severely limits the efficiency of photo-induced alkyl radical therapy. In this context, a synergistic antitumor platform named MoS_2_@AIBI-PCM nanoflowers was developed [25]. The polyethylene-glycol-functionalized molybdenum disulfide (PEG-MoS_2_) facilitated GSH depletion and improved the therapeutic efficacy.

#### 2.1.5. Light-Enhanced Fenton/Fenton-like Reaction

Arising from the chain reaction between two valence iron (Fe^2+^) and hydrogen peroxide (H_2_O_2_), Fenton reaction could generate hydroxyl radicals (·OH) with significantly higher oxidation performance than ^1^O_2_ (E(·OH/H_2_O) = 2.80 V, E(^1^O_2_/H_2_O) = 2.17 V) [26]. The typical Fenton reaction scheme is as follows: Fe^2+^ + H_2_O_2_ → Fe^3+^ + ·OH + OH^−^. It has been recognized that ultraviolet/visible light irradiation could prominently improve the efficiency of Fenton reactions [27,28]. Therefore, light-enhanced Fenton reactions serve as an intriguing modality for cancer treatment, with the tumor-targeting ability endowed by light and oxygen-independent property arising from the Fenton reactions. To facilitate deep-seated tumor treatment, the light wavelength can be transformed to NIR using upconversion nanoparticles (UCNP) [27]. However, the mild acidity (pH 6.5–6.9) of TME seriously lowers the efficiency of light-enhanced Fenton reactions, since the suitable pH range for Fenton reaction is 3-4 [29]. Aside from pH value, the low H_2_O_2_ amount in the tumor microenvironment also hampers the efficiency of light-enhanced Fenton reactions. Currently, to boost the efficiency of light-enhanced Fenton reactions, the following approaches have been applied and demonstrated to be effective, including (1) light-induced temperature elevation [30,31,32,33], (2) decreasing tumor environment pH [34], (3) increasing tumor H_2_O_2_ level [35], and (4) development of Fenton-like reactions that have better efficiency at neutral or mildly acidic pH [36,37].

**Figure 4:**
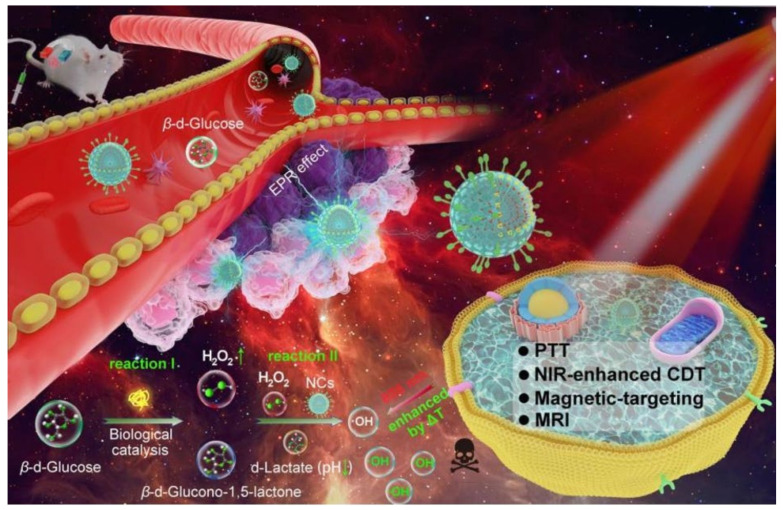
Schematic illustration of H_2_O_2_, pH, and photothermal-enhanced Fenton reaction based on γ-Fe_2_O_3_-GOx-DMSN for hypoxic tumor therapy. Adapted with permission from Ref. [32]. Copyright 2020 American Chemical Society.

For instance, Dong et al. prepared a nanoplatform containing γ-Fe_2_O_3_ and natural glucose oxidase (GOx) (Figure 4) [32]. The γ-Fe_2_O_3_ served as both photothermal therapy (PTT) agent and Fenton catalyst. Upon 808 nm light irradiation, the PTT-induced temperature elevation remarkably improved the Fenton reaction efficiency. Moreover, the GOx could significantly consume glucose in the tumor cells, resulting in pH decrease and H_2_O_2_ generation, facilitating the Fenton reaction. This research highlighted the great potential of the phototherapy combining PTT, Fenton reaction, and glucose starvation therapy.

Light-enhanced Fenton-like reactions that have high efficiency at neutral or slightly acidic conditions were also investigated for hypoxic tumor therapy. For example, Wang et al. prepared Cu_2_Se hollow nanocubes which have excellent Fenton-like properties at neutral pH, and as well high NIR II (1000−1350 nm) photothermal conversion efficiency [38]. Under NIR II light irradiation, the photothermal effect may remarkably improve the efficiency of Fenton-like reactions, causing efficient tumor destruction. The deep tissue penetration depth of NIR II light is beneficial for the treatment of deep-seated tumors.

#### 2.1.6. Type I PDT

**Figure 5:**
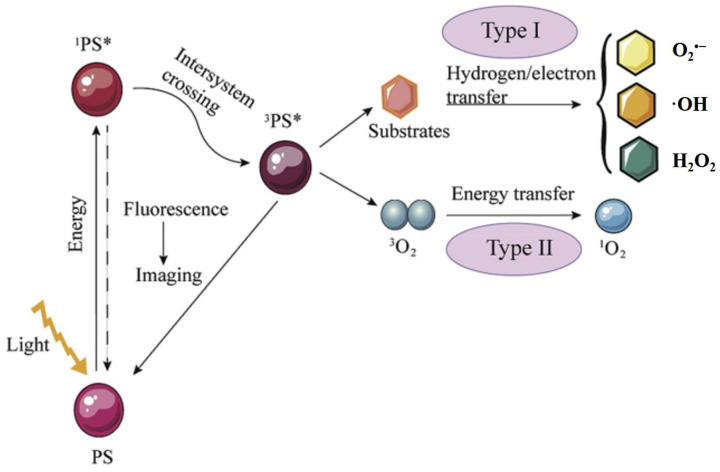
Schematic illustration of the mechanism of type I and type II photodynamic therapy. PS, photosensitizer. Adapted with permission from Ref. [5]. Copyright 2020 Elsevier.

When excited by light, a photosensitizer converts from the ground state to an unabiding singlet state (^1^PS*). This transient singlet state has two alternative routes [5]. Firstly, the singlet state is transformed back to the ground state accompanying fluorescence (which facilitates tumor imaging) emission or heat generation; secondly, the singlet state is converted into the excited triplet state (^3^PS*) with a long lifetime, which could undergo type I or type II reaction pathways [39]. In most cases, photosensitizers generate ROS via type II process, where oxygen is indispensable. In this process, the photosensitizers in the excited triplet state transfer energy to molecular oxygen (^3^O_2_), resulting in generation of ^1^O_2_, a type of ROS. Nevertheless, in the type I process, ROS including superoxide anion (O_2_^•−^) and ·OH, are produced by transferring electron/hydrogen when photosensitizers interact with substrates (biomolecules). Therefore, type I PDT is oxygen-independent. In tumor PDT, the cytotoxic ROS leads to cancer cell death. The mechanism of type I and type II PDT is presented in Figure 5.

Because of the oxygen-independent property of type I PDT, a variety of materials were designed and prepared to regulate the PDT mechanism and enhance type I PDT [40,41,42,43,44,45,46,47]. It is recognized that for the majority of photosensitizers, type I and II pathways typically exist simultaneously but competitively [48,49,50,51]. Till now, full understanding of the mechanism of transformation from type II to type I mechanism is still a great challenge. However, recent studies suggest that the electronic environment around the photosensitizer, or the electron donor groups, may play an important role in the evolution from type II to type I mechanism. It has been demonstrated that the Type I photochemical mechanism of various photosensitizers, can be enhanced via (1) physical encapsulation and (2) chemical modification [52,53,54]. For instance, Ding et al. studied the effect of the micelle carrier microenvironment on the photophysical features of a photosensitizer 5,10,15,20-tetrakis(*meso*-hydroxyphenyl)porphyrin (mTHPP) [55]. Electron-rich poly(2-(diisopropylamino)ethyl methacrylate) (PDPA) micelle encapsulation increased type I reactions generating superoxide radical anions, whereas the electron-deficient poly(D,L-lactide) micelle encapsulation produced ^1^O_2_ as predominant species by type II mechanisms. Consequently, the electron-rich PDPA micelle encapsulation increased the ability to kill cancer cells under aerobic conditions. Similarly, a recent research encapsulated a synthesized photosensitizer boron difluoride dipyrromethene (BODIPY)-vadimezan conjugate (BDPVDA), into an electron-rich amphiphilic polymer methoxy-poly(ethylene glycol)-b-poly(2-(diisopropylamino) ethyl methacrylate) (mPEG- PPDA), to obtain PBV NPs [56]. The PBV NPs achieved highly efficient type I PDT even under extremely hypoxic conditions (2% O_2_) because of its extraordinary core–shell intermolecular electron transfer. Moreover, Li et al. prepared a novel nanodot by self-assembly of 2,4,6-tris-(*N*,*N*-dimethylaminomethyl)phenoxy substituted zinc(II) phthalocyanine, PcA [57] for highly efficient type I photoreactions. The PcA nanodots having amino groups showed high superoxide anion generation efficiency, whereas its counterpart without amino groups generated no superoxide anion. This suggested the significant role of electron donor groups in the enhancement of type I mechanism. Nevertheless, the mechanism of transformation from type II to type I pathway may be complicated, and needs to be further studied.

In addition to the abovementioned modalities, oxygen-independent photo-induced carbon monoxide generation [58] were also developed to address hypoxia in PDT.

### 2.2. Oxygen-Economizing PDT

#### 2.2.1. Mitochondria Inhibition

**Figure 6:**
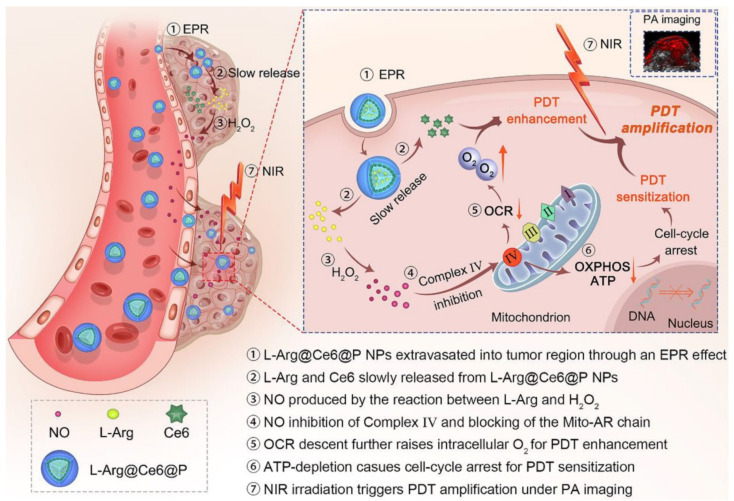
NO-based theranostic nanocomposite which enhances PDT efficiency via economizing oxygen and impeding ATP synthesis. EPR, enhanced permeability and retention. OXPHOS, oxidative phosphorylation. ATP, adenosine triphosphate. NIR, near-infrared. PA, photoacoustic. Reprinted with permission from Ref. [79]. Copyright 2021 Ivyspring International Publisher.

Very recently, increasing attention has been drawn to the inhibition of the oxidative phosphorylation (OXPHOS) pathway in mitochondria of cancer cells in order to economize oxygen for PDT reactions. Although tumors acquire energy predominantly via the “Warburg effect” [59], seriously accelerated OXPHOS was still observed in considerable malignant cell lines during tumorigenesis, development, and metastasis [60,61]. Consequently, diverse mitochondria respiratory chain inhibitors, including α-tocopherol succinate [62], papaverine [63], atovaquone [64,65,66,67,68], tamoxifen [69,70], metformin [71,72,73,74,75,76,77], and nitric oxide (NO) [78,79,80,81], have been used for reducing oxygen consumption and increasing PDT efficiency. For example, a nanoplatform was prepared through incorporating L-Arginine and a photosensitizer chlorin e6 with poly-lactic-co-glycolic acid (PLGA) (Figure 6) [79]. The L-Arginine could be transformed to NO after reaction with the over-expressed H_2_O_2_ in the tumor. The NO inhibited the mitochondrial Complex Ⅳ (Cytochrome C oxidase) and reduced oxygen consumption to increase PDT efficiency. The inhibition of the mitochondrial Complex Ⅳ also retarded adenosine triphosphate (ATP) production, which could sensitize the tumor cells for PDT.

#### 2.2.2. Fractional PDT

Tumor hypoxia is exacerbated during type II PDT process due to the consumption of oxygen by type II PDT reactions. Fractional PDT uses fractionated (i.e., intermittent) light irradiation instead of continuous light irradiation to activate the photosensitizer, allowing for oxygen replenishment during breaks between irradiation periods. Fractional PDT makes full use of the oxygen available. Many studies suggest that fractional PDT may improve treatment efficiency [82,83,84,85]. In addition to the modification of irradiation protocols, efforts have been made towards improvement of photosensitizers in fractional PDT. To further enhance fractional PDT, Turan et al. incorporated boron-dipyrromethene (BODIPY) with 2-pyrdidone to obtain a novel photosensitizer named PYR6 [83]. Upon light irradiation (light cycle), PYR6 generated ^1^O_2_, some of which was absorbed by reaction with 2-pyridone to form the corresponding endoperoxide, that is, EPO7. When light irradiation was paused (dark cycle), EPO7 released ^1^O_2_ because of a thermal cycloreversion of the endoperoxide and regenerated PYR6. As a result, the photodynamic process could continue in the dark as well as in the light cycles. In vitro experiments demonstrated that this new fractional PDT approach based on PYR6 obviously increased PDT efficiency. In addition, multiple photosensitizer injections might also improve hypoxic tumor PDT efficiency via palliating the problem of photochemical consumption of oxygen.

### 2.3. Oxygen-Supplementing PDT

Oxygen-supplementing PDT provides the advantage that it could attenuate hypoxia-related issues such as drug-resistant gene expression. The methods for supplementing oxygen in tumor PDT can be categorized mainly into six routes. The merits and limitations of these routes are summarized in Table 1.

#### 2.3.1. Increasing Oxygen Utilization Efficiency Using Micro-/Nanomotors

The employment of synthetic micro-/nanomotors is an emerging strategy for overcoming tumor hypoxia and improving PDT [86,87,88,89]. Micro-/nanomotors are micro-/nano particles which can convert different energy (chemical energy or other external energy resources including light, electronic, and magnetic fields) to propulsion for autonomous movement. Because of their movement property, these motors may increase the oxygen utilization efficiency and boost hypoxic tumor PDT via at least two pathways: (1) The motion characteristic of motors facilitates blood vessel and tissue penetration [90,91]. This is beneficial for the penetration of oxygen and photosensitizers to the central hypoxic aeras of tumors, leading to inhibition of tumor metastasis and more effective tumor destruction. (2) These motors may promote the diffusion of oxygen and photosensitizers inside tumor cells, resulting in expanded distribution of generated ROS in the tumor site. This may overcome the PDT deficiency caused by the limited diffusion range of ROS, and consequently lead to simultaneous destruction of multiple important organelles, resulting in accelerated cancer cell death.

**Figure 7:**
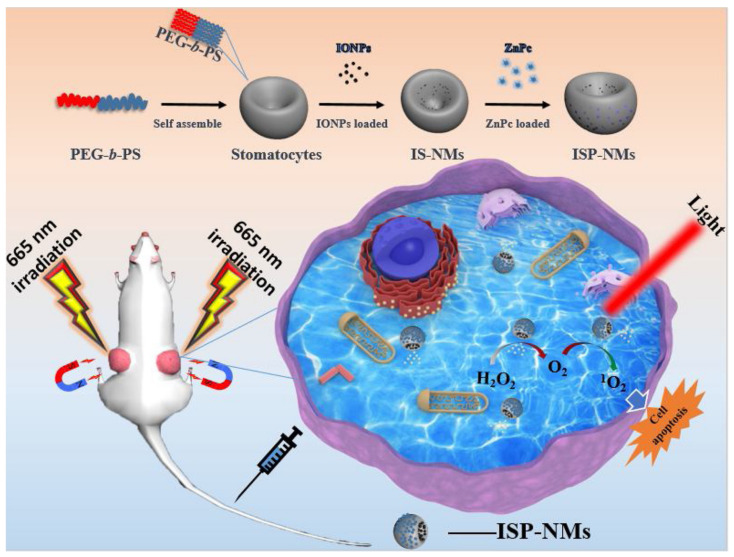
Preparation of ISP-NMs and mechanism of nanomotor-mediated photodynamic therapy. PEG-*b*-PS, poly(ethylene glycol) block polystyrene. IONP, iron oxide nanoparticle. ZnPc, zinc phthalocyanine. Reprinted with permission from Ref. [82]. Copyright 2020 Elsevier.

For instance, recently Zhang et al. constructed a nanomotor composed of self-assembly stomatocyte-like structure of poly(ethylene glycol) block polystyrene (PEG-*b*-PS), iron oxide nanoparticles (IONPs) and the photosensitizer zinc phthalocyanine (ZnPc) (Figure 7) [86]. The as-prepared nanomotor was denoted as ISP-NMs. The stomatocyte-like structure conferred the system movement function. Because of magnetism of IONPs, the nanomotors could accumulate effectively in tumor sites under magnetic field guidance. After reaching the tumor, IONPs could decompose tumor endogenous H_2_O_2_ to form oxygen as propelling force for ISP-NMs movement. Fluorescence images showed that the oxygen-propelled movement prominently enlarged the intracellular distribution of photosensitizers. Accordingly, the movement of ISP-NMs enhanced PDT efficiency. In vitro studies on Hela cells showed that ISP-NMs killed more cancer cells than ZnPc&IONPs, and in vivo studies on tumor-bearing mice demonstrated that ISP-NMs suppressed tumor growth more remarkably than ZnPc&IONPs.

For another instance, Gao et al. prepared a red-blood-cell-mimicking (RBCM) micromotor that could actively transport oxygen and photosensitizer for improved PDT [87]. The RBCM micromotors contain Fe_3_O_4_ nanoparticle-loaded hemoglobin, photosensitizer indocyanine green (ICG), and red blood cell membranes. When exposed to an acoustic field, the RBCM micromotors could convert the acoustic energy to movement at a velocity of up to 56.5 μm s^−1^ in biological media. The direction of the movement could be navigated by an external magnetic field. The motion characteristic and oxygen and photosensitizer-carrying ability of the RBCM micromotors make them promising agents for future PDT.

In a word, micro-/nanomotors could realize a novel “active PDT” with improved anticancer effect. The motion characteristic of active PDT may increase the utilization efficiency of oxygen and facilitate tumor hypoxia alleviation for enhanced PDT.

#### 2.3.2. Living Organism Oxygen Generators

The employment of photosynthesis of microorganisms or chloroplast components extracted from green plants, is an emerging and promising approach to increase tumor oxygen concentration and improve PDT.

In the relatively early stage (since Year 2019), researchers utilized several kinds of photosynthetic microorganisms, including Chlorella [92], Cyanobacteria [93,94,95,96,97,98], and *Spirulina* [99], to achieve tumor oxygenation and PDT enhancement under visible light irradiation. After administration into the tumor-bearing mice, these microorganisms (usually modified with chemicals) showed prominent PDT efficiency enhancement via tumor hypoxia alleviation. The co-loaded artificial drugs [95], or innate chlorophyll released from the microorganism [99], served as photosensitizers. For instance, Zhou et al. constructed an autotrophic light-triggered green affording-oxygen engine (ALGAE) consisting of Chlorella and calcium alginate [92]. In vitro and in vivo results showed that the PDT efficiency was remarkably enhanced due to the photosynthesis of Chlorella. Moreover, the ALGAE may suppress tumor metastasis by downregulating the expression of vascular endothelial growth factor and HIF-1α, and upregulating the expression of epithelial cadherin. Calcium alginate protected the Chlorella from the phagocytosis of macrophage, allowing for its long period existence in the tumor site, and consequently highly efficient repetitive PDT treatments. However, these studies had to employ visible light to activate PDT, due to the requirement of the natural photosynthesis. This limits the application on deep-seated tumors.

More recently, microorganisms have been combined with UCNPs to move the activation light wavelength to the NIR range. Zhang et al. used Cyanobacteria as a living carrier for photosensitizer chlorin e6 conjugated UCNP to build a self-supplying oxygen PDT system [100]. Under NIR irradiation, this system relieved tumor hypoxia and boosted PDT efficiency both in vitro and in vivo. The recent progress on the photosynthetic microorganism-enhanced PDT also includes the combination of microorganism and perfluorocarbons for oxygen production and collection [101], and improvement of biocompatibility via biomineralization [102].

**Figure 8:**
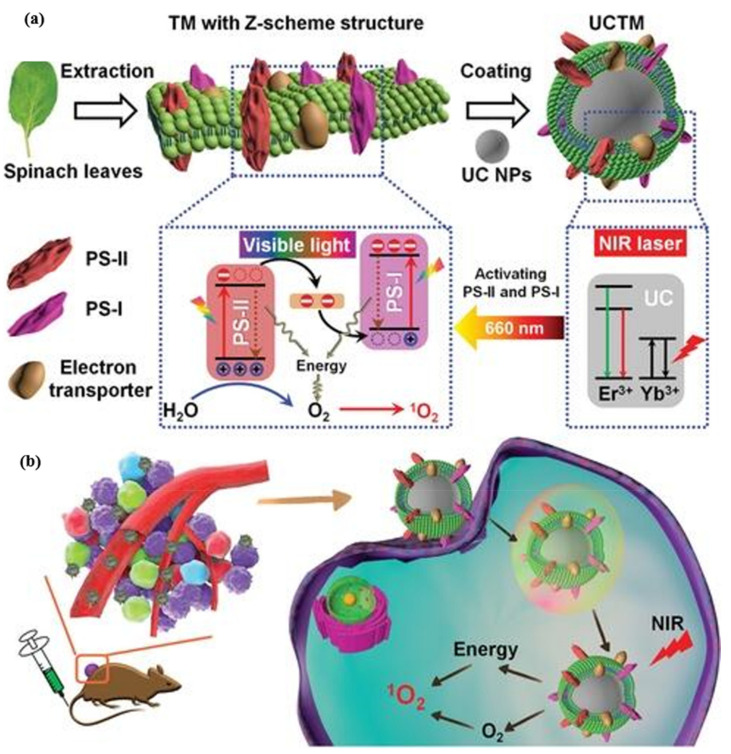
Illustration of thylakoid membrane-decorated upconversion nanoparticles (UCTM) for enhanced photodynamic efficacy against hypoxic tumors. (**a**) Preparation of UCTM and the corresponding electron transfer as well as oxygen and reactive oxygen species generation mechanism of UCTMs under near-infrared light irradiation. (**b**) the therapeutic process mediated by synchronous oxygen supply and reactive oxygen species generation under near-infrared light irradiation. Reprinted with permission from Ref. [104]. Copyright 2021 John Wiley and Sons.

In addition to photosynthetic microorganisms, chloroplast components extracted from green plants were also used for tumor oxygen concentration elevation. Green plants rely on their chloroplasts to perform photosynthesis and generate oxygen. The oxygen generation by chloroplasts originates from the subtle Z-scheme structure containing the photosystem-I (PS-I) and photosystem-II (PS-II) located in thylakoid membranes (TM) of chloroplasts. The early research fused the thylakoid membrane with synthetic nanoparticles for efficient oxygen generation under 660 nm visible light irradiation [103]. In vivo studies demonstrated that the PDT efficiency was improved via photosynthesis-mediated tumor oxygenation. More recently, Cheng et al. prepared TM-decorated UCNPs (UCTM), realizing 980 nm NIR-activated PDT (Figure 8) [104]. The chlorophyll in the TM served as photosensitizer. Importantly, this outstanding design facilitated the synchronous and short-distance oxygen supply and ROS generation, making full use of the oxygen generated and improving PDT efficiency.

In summary, as a new strategy for tumor hypoxia alleviation, biosynthetic living organism-based oxygen supplementation provides advantages over other hypoxia-addressing strategies, but also has its own shortcomings (Table 1). The advantages include: (1) Simultaneous oxygen supply and ROS production improving PDT efficiency. The synchronization of these two processes is important for the PDT efficiency. If oxygen is supplied early before or long past light-triggered ROS generation, these oxygen molecules would be remarkably consumed by the rapid-growing tumor cells instead of the PDT reaction. This renders the PDT ineffective. Using the biological photosynthesis, the oxygen generation and conversion of oxygen to ^1^O_2_ happen at the same time when light is shone, promoting PDT efficiency. (2) Abundance of water in tumor tissues for oxygen formation via photosynthesis. Water is an essential component of living creatures, and exists in a large amount in tumors. This may facilitate oxygen production. However, this biological photosynthesis-based strategy also possesses disadvantages, including (1) Harsh TME lowering microorganism activity. As living organism or structures extracted from living organisms, the actuators of this strategy may confront difficulties in exerting or maintaining their photosynthetic activity in the harsh TME, including acidic pH, overproduced H_2_O_2_, excessive glutathione, and temperature. For instance, the temperature of the tumor tissues may limit the photosynthetic activity of TMs extracted from green plants, since the activity of photosynthetic systems in green plants is very sensitive to temperature elevation [105,106]. (2) Large size hindering deep tumor penetration. The usual size of microorganisms is several micrometers. The large size might hinder the delivery of these microorganisms through the capillaries (the tiniest being 4–5 mm in diameter) to the tumor [107]. Moreover, in comparison with typical nanomaterials with diameters below 100 nm, the microorganisms may have lower ability to penetrate to the hypoxic tumor core [108], resulting in insufficient tumor oxygenation and inadequate PDT efficacy. (3) Light wavelength hampering deep-seated tumor treatment. The wavelengths of the light for the photosynthesis lie in the range of visible light, which limits the deep-seated tumor treatment. To sum up, the biological photosynthesis strategy is a promising approach for hypoxic tumor PDT, and its performance could be further improved. The impact of these microorganisms on the human body, such as their effect on the immune system, could also be further explored.

#### 2.3.3. Light-Driven Water Splitting

Chloroplasts in green plants absorb solar energy and transfer it to water to form oxygen. Inspired by this phenomenon, diverse water-splitting nanocomposites including organic, inorganic, or hybrid materials have been prepared for oxygen generation and PDT enhancement. This strategy has unique advantages including synchronous activation of oxygen supply and ROS generation, and abundance of water in the human body for oxygen production.

C_5_N_2_ nanoparticles [109], C_3_N_4_ [110,111], tungsten nitride [112], carbon nanodot [113], graphdiyne oxide [114], iron disulfide (FeS_2_) [115], cobalt phytate [116], etc., have been used in the water-splitting strategy. C_3_N_4_-related materials have been intensively studied for the enhancement of PDT via water splitting-mediated oxygen generation. Recent progress on C_3_N_4_-related nanostructures include the enhancement of light absorption at 700–900 nm optical transparency window [117] and utilization of two-photon excitation [118], which facilitate deep-seated tumor treatment.

#### 2.3.4. Modification of Tumor Blood Circulation

It was reported that pre-administration of heparin, a clinically utilized anticoagulant, could temporarily prevent vessels from shutting down during PDT treatment, enhancing oxygen supply and PDT efficiency [119]. The oxygenation also improved light delivery because it maintained the tissue optical properties during the PDT treatment procedure. It was also demonstrated that a warm water bath (43 °C) could improve the blood flow velocity and increase tumor oxygen level, boosting PDT [120].

#### 2.3.5. Tumor H_2_O_2_ Decomposition

The level of H_2_O_2_ in the tumor is higher than normal tissues owing to the higher H_2_O_2_ generation rate of cancer cells (up to 0.5 nmol/10^4^ cells/h) [121,122]. The overproduced H_2_O_2_ is related to the angiogenesis, proliferation and metastasis of tumors [3]. Therefore, decomposing the tumor endogenous H_2_O_2_ to produce oxygen is a promising approach for tumor hypoxia alleviation and PDT enhancement. This approach has been intensively studied, and a wide variety of nanocomposites capable of promoting H_2_O_2_ decomposition were fabricated and demonstrated effective for PDT enhancement. The materials that could accelerate H_2_O_2_ decomposition mainly include metal-based materials and catalase.

Recently, the employment of metal-based materials to promote the decomposition of tumor endogenous H_2_O_2_ to generate oxygen has attracted increasing attention. Titanium dioxide-modified palladium (Pd) nanosheets [123], Pd nanoparticles [124], defective cobalt (Co) hydroxide [125], Co_3_O_4_ nanoparticles [126], iridium (Ir) oxide [127,128], cerium (Ce) oxide nanoparticles [129,130], platinum (Pt) nanozyme [131,132], gold (Au) nanoclusters [133], iron (Fe)-based materials [134,135,136,137,138], etc. have been used to accelerate the decomposition of H_2_O_2_ to generate oxygen. For instance, recently, Xu et al. developed a defective cobalt hydroxide-based nanoagent for hypoxia-ameliorating photothermal-enhanced photodynamic therapy [125]. They converted cobalt hydroxide into black and defect-abundant nanosheets via oxidation, extending the absorbance range to NIR-I (750−980 nm), and achieving high photothermal conversion efficiency and enhanced H_2_O_2_ decomposition activity. The defective cobalt hydroxide nanosheets were further linked to chlorin e6 to form a nanoagent denoted as BCS-Ce6. In vivo experiments showed that BCS-Ce6 could almost eliminate the implanted tumors in mice. This research provided an innovative approach to produce novel therapeutic nanostructures for TME-modulating phototherapy.

Among the metal-based materials for generating oxygen through H_2_O_2_ decomposition, MnO_2_ is a widely studied material [139,140,141,142,143,144,145,146,147,148,149]. MnO_2_ could produce oxygen and Mn^2+^ at acidic pH, particularly in the presence of H_2_O_2_. It was also found that MnO_2_ could decrease the intratumoral glutathione level. These properties make MnO_2_ highly responsive to the TME featured with acidic pH, overproduced H_2_O_2_ and abundant glutathione, which is beneficial for tumor selectivity. Moreover, the generated Mn^2+^ can enhance T1-weighted magnetic resonance imaging, allowing for imaging-guided tumor therapy. However, the generated Mn^2+^ may elicit toxicity if its concentration is high.

Catalase is an important enzyme in the human body. It catalyzes the transformation of H_2_O_2_ to water and oxygen. It is widely used in the construction of nanosystems for hypoxic tumor PDT [150,151,152,153,154]. For instance, catalase was co-encapsulated with a photosensitizer MBDP and a chemotherapeutic drug doxorubicin to obtain FA-L@MD@CAT [155]. The FA-L@MD@CAT could catalyze tumor endogenous H_2_O_2_ to generate oxygen, enhancing cancer chemo-photodynamic therapy. Importantly, examinations on tumor-bearing mice showed that FA-L@MD@CAT helped to reverse M2-polarized tumor associated macrophages (TAMs), and increased cytotoxic T lymphocyte (CTL) levels and CD8+ CTL / regulatory T cell (Treg) ratio. This suggested that FA-L@MD@CAT could trigger immunogenic cell death (ICD) to induce CTL infiltration into tumors, and reverse immunosuppressive TME by addressing tumor hypoxia. This research provided a promising nanoplatform for enhanced cancer chemo-photodynamic therapy, and highlighted the important role hypoxia-addressing PDT may play in the enhancement of antitumor immune response. Recently, genetically modified *Escherichia coli* (*E. coli*) with overexpressed human catalase was combined with photosensitizers such as chlorin e6 [156] and black phosphorus quantum dots [157], and enhanced PDT by catalyzing H_2_O_2_ into O_2_ in the tumor site.

The metal-based materials and catalase have their distinct strengths and limitations for tumor H_2_O_2_ decomposition (Table 2). Metal-based materials (for instance, Co_3_O_4_ [126]) may have better stability than natural catalase when they are exposed to solutions with different pH values and temperatures. However, the activity of metal-based materials is lower than catalase [158], and the generated metal ions may cause toxicity if their concentrations are high. In contrast, catalase, as a biological catalyst, has higher activity than metal-based materials. Nevertheless, the enzymatic activity of catalase is easily compromised in harsh environments such as improper pH values and temperatures. The activity may also be hampered by chemical modification during preparation of nanocomposites, since the enzymatic activity relies on the correct folding of the peptides [159].

The tumor H_2_O_2_ decomposition strategy has distinct advantage of high tumor specificity, as H_2_O_2_ is overproduced in the tumor site. However, the efficiency of this strategy is limited by the low H_2_O_2_ level in the tumor (50∼100 μM) [160].

#### 2.3.6. Oxygen Delivery

Because of the oxygen affinity of hemoglobin, perfluorocarbon, or metal–organic frameworks, these materials can be used to efficiently transport oxygen to the tumor site and boost PDT. The merits and shortcomings of these materials are listed in Table 3.

##### Hemoglobin and Red Blood Cell-Based Oxygen Carriers

Hemoglobin is the natural oxygen carrier residing in red blood cells. Each hemoglobin molecule can bind up to four oxygen molecules. Owing to the oxygen-carrying ability of hemoglobin, a number of hemoglobin-based nanosystems have been constructed to transport oxygen to the tumor site and enhance PDT. As a natural protein for oxygen transportation in living organisms, hemoglobin might provide the advantage that the transported oxygen can be easily released in H^+^ and carbon-dioxide-rich TME (Bohr effect) [161]. Despite its great potential, free hemoglobin faces limitations such as potential toxic effect after autoxidation. This autoxidation could be exacerbated in the H_2_O_2_-rich TME [162]. It has been reported that free hemoglobin in circulation may elicit side effects such as kidney tubule damage [163]. Therefore, hemoglobin is often further engineered for hypoxic tumor PDT through chemical conjugation or physical encapsulation [164,165,166,167].

For example, recently a paramagnetic nanostructure consisting of hemoglobin, Gd, and PEGylated chlorin e6, was prepared using a non-toxic hemoglobin-mediated biomimetic synthesis method [168]. Interestingly, hemoglobin functioned as a biological template, stabilizer for the nanoparticle formation, and substrate for the loading of chlorin e6 in the synthesis process, and maintained its ability to bind and carry oxygen. In vitro and in vivo experiments showed that the nanostructure enhanced PDT efficiency by replenishing tumor hypoxia, and could be guided by magnetic resonance imaging. The unique eco-friendly and non-toxic preparation method and the potent PDT efficiency make this nanostructure a promising nanomedicine for future application.

Hemoglobin-based nanosystems are frequently encapsulated with red blood cell membranes to increase their biocompatibility and ability to escape from elimination by immune system [169]. In addition, red blood cells can be linked with photosensitizers and other substance to realize hypoxia-alleviating PDT. For instance, Wang et al. installed photosensitizer (rose bengal) and tumor-targeting RGD peptide functionalized upconversion nanoparticles onto the surface of indocyanine green-loaded red blood cells, and obtained RBCp. The RBCp enhanced PDT efficiency and showed great potential for second near-infrared window fluorescence imaging-guided tumor surgery and photodynamic therapy [170].

##### Perfluorocarbon-Based Oxygen Carriers

Perfluorocarbons are synthetic molecules consisting of carbon and fluorine atoms. They have extraordinary oxygen solubility because of the high electronegativity of fluorine [11]. At normal atmospheric pressure, perfluorocarbons can load about twice the amount of oxygen as blood can [171]. Moreover, perfluorocarbons exhibited excellent biocompatibility. For instance, perfluorohexane is approved by FDA. It has also been found that the lifetime of ROS can be extended in perfluorohexane [172] or perfluorotributylamine (PFTBA) [173], which enhanced PDT efficiency.

Oxygen delivery by perfluorocarbons has been extensively studied. A variety of perfluorocarbons have been used for the preparation of nanoformulations to achieve oxygen-supplementing PDT, including perfluorohexane [174,175], perfluorooctyl bromide (PFOB) [176], perfluoro-15-crown-5-ether [177], PFTBA [178], etc. Recently, a PFOB-based nanostructure was reported to combat liver metastasis of colon cancer [179]. PFOB was dispersed into porphyrin grafted lipid (PGL) nanoparticles (NPs), and then oxygen was loaded to obtain O_2_@PFOB@PGL NPs. The O_2_@PFOB@PGL NPs achieved very high loading content for both porphyrin and PFOB. It served as a prominent oxygen reservoir, and remarkably relieved tumor hypoxia. The O_2_@PFOB@PGL NPs not only dramatically improved PDT efficiency to eliminate the primary tumor, but also addressed tumor hypoxia to inhibit hypoxia-promoted colorectal cancer liver metastasis via decreasing COX-2 expression. Additionally, the O_2_@PFOB@PGL NPs could act as a contrast agent for fluorescence and computed tomography imaging, facilitating bimodal imaging-guided PDT. To sum up, this nanostructure provides a promising strategy to combat colon cancer and to inhibit metastasis by replenishing tumor hypoxia.

##### Metal–Organic Frameworks

Recently, metal–organic frameworks (MOFs) were used to transport oxygen and enhance PDT against hypoxic tumors. MOFs are materials that are prepared via coordinating metal ions with organic linkers. Owing to the large surface area and uniform pore size, MOFs have excellent ability of gas storage and separation. Gao et al. used the MOF UiO-66 as oxygen reservoir for enhanced PDT against hypoxic tumors [180]. The UiO-66 was combined with the photosensitizer ICG, and encapsulated inside red blood cell membranes to obtain a nanoplatform O_2_@UiO-66@ICG@RBC. The oxygen transportation by the UiO-66 prominently boosted the PDT efficacy. Moreover, the red blood cell coating endowed the O_2_@UiO-66@ICG@RBC ability to escape from elimination by immune systems, further improving antitumor effect. Xie et al. used another MOF material, Zeolitic imidazolate framework-90 (ZIF-90), as oxygen reservoir to improve PDT efficacy [181]. They prepared a multifunctional system UC@mSiO_2_-RB@ZIF-O_2_-DOX-PEGFA (URODF) for combined PDT and chemotherapy. The upconversion nanoparticles (UC) could convert NIR irradiation to ultraviolet/visible light and activate the photosensitizer Rose Bengal (RB) loaded on the mesoporous silica shell (mSiO_2_). Doxorubicin (DOX) and NH_2_-PEG modified folic acid (PEGFA) were included for chemotherapy. In vitro and in vivo results indicated the outstanding therapeutic outcome of URODF.

The inherent multifunctionality confers MOFs unique advantage over other oxygen carriers. For instance, MOFs are good carriers for photosensitizers and chemotherapeutic drugs. However, the metal ions might elicit safety concerns.

## 3. Emerging Trends and Outlook

Oxygen-independent phototherapy, oxygen-economizing PDT, and oxygen-supplementing PDT have their unique advantages and limitations, as summarized in Table 4. Therefore, creative combination of these strategies might bridge advantages and eliminate shortcomings, advancing PDT treatment efficacy. Antitumor efficiency is an important index for evaluating hypoxia-addressing strategies. However, the antitumor efficiency depends on multiple factors, including what photosensitizer is used, the loading efficiency of photosensitizer, light irradiation intensity, light irradiation dose, etc. Therefore, it seems impossible to conclude which hypoxia-addressing strategy has highest antitumor efficiency. However, the antitumor efficiency of each research, that is, the efficiency of each special micro/nanosystem, can be roughly compared according to the volume change of mouse tumors after treatment with the micro/nanosystems. Nowadays, generally speaking, decelerated tumor growth in comparison with the control group can be considered effective for tumor treatment, but not satisfactory; decrease in tumor volume till final complete elimination of the tumor in 1 or 2 weeks may represent high antitumor efficiency; complete tumor elimination within 10 minutes, which was realized by the photogenerated hole therapy based on SA-TCPP as introduced in this review, can be viewed as exceedingly high antitumor efficiency.

It is noteworthy that by addressing hypoxia, remarkable achievements on cancer treatment have been made, including potent efficacy (tumor elimination within 10 min), inhibition effect on tumor metastasis, and promotion of antitumor immune response. With these encouraging achievements, we believe that considerable improvement of cancer treatment outcome could be realized by advanced hypoxia-addressing materials. Meanwhile, the continuous emergence of creative strategies, such as the employment of micro-/nanomotors, microorganism-mediated oxygen generation, and utilization of ROS with stronger oxidation performance than ^1^O_2_ (e.g., OH), will inspire more novel and useful strategies for PDT against hypoxic tumors.

We could also conclude that the temporal and spatial control is important for the improvement of PDT efficiency. It is noteworthy that synchronizing oxygen supply and photosensitizer activation, shortening the distance between oxygen and photosensitizer molecules, shortening the distance between ROS and biomolecules such as mitochondria DNA molecules, and enlarging the diffusion distances of oxygen and photosensitizers in tumor tissues, can improve PDT efficacy. The extremely short lifetime (less than 200 ns) and diffusion distance (about 20 nm) of ROS severely limit PDT efficiency. Therefore, increasing the lifetime of ROS could lead to enhancement of PDT.

Despite the bright prospect of hypoxia-addressing PDT, challenges for the development remain. For example, the effect of hypoxia amelioration on the tumor metastasis inhibition and antitumor immunity needs to be further studied and improved. Although hypoxia-activable chemotherapeutic drugs have been combined with photosensitizers, chemicals that can only show toxicity in the presence of both hypoxia and light, remain to be investigated.

## Figures and Tables

**Table 1:** A summary of the representative materials, strengths, and shortcomings of different approaches for oxygen-supplementing PDT. ROS, reactive oxygen species.

Strategy	Representative Materials	Strengths	Shortcomings
Increasing oxygen utilization efficiency using micro-/nanomotors	Poly(ethylene glycol) block polystyrene, Fe_3_O_4_ nanoparticle-loaded hemoglobin	Deep tumor penetration through movement	Maximum efficiency limited by oxygen concentration
Living organism oxygen generators	Chlorella, Cyanobacteria, *Spirulina*, and thylakoid membrane of green plants	Synchronous activation of oxygen supply and ROS generation, abundance of water in the human body beneficial for oxygen generation	Harsh tumor microenvironment harming organism activity; micrometer size limiting deep tumor penetration; light wavelengths in visible light range
Light-driven water splitting	Tungsten nitride, carbon nanodot, graphdiyne oxide, iron disulfide, cobalt phytate, C_3_N_4_	Synchronous activation of oxygen supply and ROS generation, abundance of water in the human body for oxygen production	Safety concern due to presence of metal ions
Modification of tumor blood circulation	Heparin, warm water bath	Concurrent improvement of light delivery	Weak effect on tumor regions distant from blood vessels
Tumor H_2_O_2_ decomposition	Metal-based materials, catalase	Inherent tumor specificity	Efficiency limited by H_2_O_2_ concentration
Oxygen delivery	Hemoglobin, perfluorocarbon, metal–organic frameworks	High efficiency	Lack of inherent tumor specificity

**Table 2:** A summary of the strengths and shortcomings of different materials for tumor H_2_O_2_ decomposition.

Materials for Tumor H_2_O_2_ Decomposition	Strengths	Shortcomings
Metal-based materials	Superior stability in environments with different pH values and temperatures	Lower activity than catalase, safety concern arisen from metal ions
Catalase	High activity as a biological catalyst	Low stability in environments with different pH values and temperatures

**Table 3:** A summary of the representative materials, strengths, and shortcomings of different oxygen delivery approaches for hypoxic tumor PDT. ^1^O_2_, singlet oxygen. ZIF-90, zeolitic imidazolate framework-90.

Strategy	Representative Materials	Strengths	Shortcomings
Hemoglobin	Modified hemoglobin, red blood cells	Tumor-specific oxygen release owing to Bohr effect, ability of the red blood cell membrane to escape from immune clearance	Low oxygen loading capacity, safety concern
Perfluorocarbon	Perfluorohexane, perfluorooctyl bromide, perfluoro-15-crown-5-ether, perfluorotributylamine	High oxygen loading capacity, FDA-approved materials such as perfluorohexane, increased ^1^O_2_ lifetime	Relatively weak tumor-specific oxygen release
Metal–organic frameworks	UiO-66, ZIF-90	Multifunctionality, high oxygen loading capacity	Potential toxicity arisen from metal ions

**Table 4:** An overview of strategies for modulating tumor hypoxia in photodynamic therapy. ROS, reactive oxygen species. ^1^O_2_, singlet oxygen. ATP, adenosine triphosphate.

Strategy	Strengths	Shortcomings
Oxygen-independent phototherapy	Generation of ROS with stronger oxidation performance than ^1^O_2_	Hypoxia-related issues such as drug-resistant gene expression unresolved
Oxygen-economizing PDT	Synergetic ATP production inhibition	Maximum efficiency limited by the existing oxygen content
Oxygen-supplementing PDT	Hypoxia-related issues such as drug-resistant gene expression attenuated	Difficulty in achieving both continuous and efficient oxygen supply

## Data Availability

Not applicable.
